# Baseline Predictors of Adverse Outcomes for Transthyretin Amyloidosis Cardiomyopathy Patients Treated and Untreated with Tafamidis: A Canadian Referral Center Experience

**DOI:** 10.3390/jcm13185490

**Published:** 2024-09-16

**Authors:** Karan Shahi, Robert J. H. Miller, Steven Dykstra, Yuanchao Feng, Jonathan G. Howlett, Victor Jimenez-Zepeda, Jan Veenhuyzen, James A. White, Nowell M. Fine

**Affiliations:** 1 Division of Cardiology, Department of Cardiac Sciences, Libin Cardiovascular Institute, University of Calgary, Calgary, AB T2N 1N4, Canada; karan.shahi@ucalgary.ca (K.S.); robert.miller@ahs.ca (R.J.H.M.); dykstras@ucalgary.ca (S.D.); yuanchao.feng@albertahealthservices.ca (Y.F.); jonathan.howlet2@albertahealthservices.ca (J.G.H.); jveenhuy@ucalgary.ca (J.V.); jawhite@ucalgary.ca (J.A.W.); 2 Division of Hematology, Department of Internal Medicine, Cumming School of Medicine, University of Calgary, Calgary, AB T2N 1N4, Canada; victor.zepeda@albertaheathservices.ca

**Keywords:** amyloidosis, transthyretin, cardiomyopathy, tafamidis, mortality, hospitalization, outcome-free survival, predictors

## Abstract

**Background:** Tafamidis is a costly therapy that improves outcomes for patients with transthyretin amyloidosis cardiomyopathy (ATTR-CM), although significant knowledge gaps exist for predicting longer-term response to treatment. The purpose of this study was to examine baseline predictors of adverse outcomes and their association with tafamidis treatment in comparison with those untreated in a clinical cohort from a Canadian ATTR-CM referral center. **Methods:** Patients with a confirmed diagnosis of ATTR-CM were included. Multivariable modeling was used to identify baseline variables associated with the primary outcome of all-cause mortality and secondary outcomes of cardiovascular mortality or hospitalization. Cox proportional hazard and competing risk analyses were used, with tafamidis modeled as a time-varying covariate. **Results:** In total, 139 ATTR-CM patients were included, with a median age of 80.9 years [74.3–86.6 years], from 2011 to 2022. The mean follow-up was 2.9 ± 1.8 years. Eighty (55%) patients were treated with tafamidis. All-cause mortality and cardiovascular mortality alone were associated with the following baseline variables: age, clinical frailty scale, systolic blood pressure, renal function, and right ventricular size and function (all *p* < 0.05), with no identified interactions with tafamidis treatment. Only baseline renal function was associated with cardiovascular hospitalization (*p* < 0.05). **Conclusion:** Important baseline variables associated with adverse ATTR-CM disease outcomes included renal function, systolic blood pressure, frailty, and right ventricular size and function. The risk factors were independent of treatment with tafamidis. These findings may help improve risk stratification for determining eligibility for ATTR-CM therapies.

## 1. Background

Transthyretin amyloidosis cardiomyopathy (ATTR-CM) is caused by the misfolding of transthyretin (TTR) proteins, leading to the formation of fibrillar amyloid deposits within the myocardial interstitium [1,2,3]. It is described as an underrecognized cause of heart failure (HF) in the community, marked by heightened morbidity and mortality [3]. ATTR-CM comprises two subtypes: hereditary (hATTR), caused by a mutation of the TTR gene, and wild-type (wtATTR), with the latter occurring in the absence of a gene mutation and predominantly affecting older individuals with a reported median onset age of >70 years [2]. 

The prognosis of ATTR-CM patients has significantly improved with the use of disease-modifying therapies [4]. Tafamidis, an oral TTR stabilizer for the treatment of wtATTR and hATTR cardiomyopathy, prevents TTR dissociation, thereby attenuating disease progression. The Tafamidis Treatment for Patients with Transthyretin Amyloid Cardiomyopathy (ATTR-ACT) study demonstrated tafamidis’ efficacy, revealing superiority over a placebo in reducing all-cause mortality and cardiovascular-related hospitalizations [5]. However, knowledge gaps persist regarding optimal use in clinical practice. While the ATTR-ACT trial demonstrated that response to therapy was significantly influenced by the baseline New York Heart Association (NYHA) functional class, other factors influencing outcome remain uncertain in both treated and untreated patients. This is of particular importance when considering not only the high cost of therapy but also the predominantly older patient population [2,6]. The goal of tafamidis therapy is to attenuate disease progression rather than improve symptoms, and the ATTR-ACT trial demonstrated an approximately 18-month duration of treatment before survival benefit was appreciated [5]. A deeper understanding of the factors impacting outcome across multiple clinical domains may facilitate an improved selection of patients most likely to benefit from treatment with this expensive medication. We, therefore, conducted a retrospective cohort study from a Canadian cardiac amyloidosis referral center to identify baseline predictors across clinical, biochemical, and cardiac imaging domains associated with major adverse events, evaluating the influence of tafamidis treatment on these outcomes in ATTR-CM patients.

## 2. Methods

A total of 139 consecutive patients with a diagnosis of ATTR-CM followed at the Cardiac Amyloidosis Clinic at the University of Calgary (Calgary, AB, Canada) between May 2011 and November 2022 were included in this retrospective, single-center cohort study. The diagnosis of ATTR-CM was confirmed using current standard diagnostic criteria, including either biopsy tissue (with proteomic analysis using mass spectrometry) or Tc-99m-PYP nuclear bone scintigraphy with SPECT following the exclusion of light chain (AL) amyloidosis [7]. Genetic testing was performed in all patients to differentiate the ATTR-CM subtype. This study was approved by the University of Calgary Research Ethics Board with a waiver of the requirement for patient-informed consent.

## 3. Data Element Collection

### 3.1. Clinical Data Collection

Clinical characteristics, including NYHA functional class, medications, electrocardiogram (ECG) and comorbidity data, biochemical laboratory values, and cardiac imaging test parameters, including cardiac magnetic resonance imaging (CMR) and transthoracic echocardiography, were extracted from the Cardiovascular Imaging Registry of Calgary (CIROC, NCT04367220), a comprehensive cardiovascular clinical outcomes registry (Libin Cardiovascular Institute, University of Calgary) [8]. The date of ATTR-CM diagnosis, defined as the date of positive Tc-99m-PYP imaging or confirmatory biopsy, was used to extract baseline data from CIROC databases for the above-mentioned variables. After obtaining baseline assessments, serial follow-up variables, as described above, were assessed at 1-year intervals from the date of diagnosis as per the standardized clinic algorithm. Any missing and/or additional data not available from CIROC were manually abstracted from patient medical records. At our center, all ATTR patients undergo routine baseline polyneuropathy screening by comprehensive neurologic evaluation at the time of diagnosis. Data regarding a diagnosis of polyneuropathy were confirmed by reviewing clinical assessment and nerve-conduction study test results and imaging studies from patient medical records, including magnetic resonance and computed tomography scans, along with polyneuropathy disability (PND) scores. The PND score stages polyneuropathy based on physical limitations of ambulation, ranging from 0 (no symptoms) to I, II, IIIa, IIIb, and IV (wheelchair-bound or bedridden) [9]. For this analysis, patients were categorized by a PND score IIIa or higher, which indicated the point where a walking aid for ambulation is required. Our center also performs routine frailty assessments for patients diagnosed with ATTR, given the typically older age of this population. Frailty was assessed using the clinical frailty scale (CFS) (range 1—very fit to 9—terminally ill) at the time of diagnosis [10]. A CFS score of ≥4 was used to define frailty for this analysis.

### 3.2. Study Outcomes

The primary outcome was all-cause mortality, and secondary outcomes included cardiovascular mortality and cardiovascular hospitalization, each considered independently. The cause of death or hospitalization was classified using international classification of diseases, tenth revision (ICD-10) codes. All outcomes were also adjudicated using patient medical records (K.S. and N.M.F).

## 4. Statistical Analyses

Continuous data that were normally distributed were expressed as the mean ± standard deviation, while those that were not normally distributed were reported as the median with interquartile range (IQR). The two-sample or paired *t*-test was performed for the comparison of groups normally distributed; otherwise, the Wilcoxon rank-sum or signed-rank tests were performed. Categorical variables were expressed as absolute frequencies and relative percentages; a comparison of these variables and changes from baseline to follow-up were performed using Fisher’s exact test or logistic regression, respectively. A two-sided *p*-value of less than 0.05 was considered statistically significant.

Univariable survival analyses were performed to determine the associations of baseline variables with the study outcome. Analyses were performed separately for each outcome: all-cause mortality, cardiovascular mortality, and hospitalization. Cox proportional hazard modeling was used for the primary outcome of all-cause mortality, while Fine–Gray competing risk regression modeling was used for the individual outcomes of cardiovascular mortality and hospitalization (with non-cardiac mortality and all-cause mortality as the competing risk, respectively). To account for the potential delay between the time of diagnosis and initiation of therapy (prior to availability), tafamidis was modeled as a time-varying covariate. Imputation was performed for variables with missing observations in the dataset, a validated method for these analyses [11]. Multivariable modeling with backward stepwise selection was then performed for each study outcome, using significant variables (*p* < 0.05) from the univariable analysis. By incorporating covariate adjustment in the multivariable models, time to treatment was corrected for in the non-randomized setting of the project. Tafamidis therapy was modeled as a time-varying covariate, which addressed immortal time bias and accounted for measurable differences between groups. Significant variables from the multivariable model were then evaluated for possible effect modification using interaction testing with tafamidis treatment. All analyses were performed using statistical software STATA Version 17, Basic Edition (College Station, TX, USA).

## 5. Results

### 5.1. Patient Characteristics and Outcomes

A total of 139 patients diagnosed with ATTR-CM (121, 87% men; median age at diagnosis [IQR] 80.9 years [74.3–86.6 years]) were included (Table 1). The hATTR subtype was present in eight (6%) patients, with the remainder having the wtATTR subtype. Twenty-two (17%) patients had aortic stenosis, 101 (73%) had atrial fibrillation or flutter, and 40 (29%) patients underwent pacemaker or implantable cardioverter-defibrillator (ICD) implantation. These values were not statistically significant when comparing those on and not on tafamidis. Among the 22 patients with aortic stenosis, 32% were classified as mild, 41% as moderate, and 27% as severe. Five of these patients underwent aortic valve replacement, with the majority receiving TAVI. At baseline, no patients had an NYHA functional class of IV; distribution across classes I–III was approximately 33%, 30%, and 37%, respectively.

Eighty (58%) patients were treated with tafamidis. A significantly higher proportion of patients on tafamidis had carpal tunnel syndrome compared to the untreated group (*p* < 0.05). Patients on tafamidis exhibited significant differences compared to untreated patients for age at diagnosis, frailty, and physical ambulation limitations. Those on tafamidis were younger, less frail, and a lower proportion required aids for ambulation (a lower proportion of patients with a PND score of ≥IIIa). Patients on tafamidis had significantly lower baseline levels of creatinine, *n*-terminal pro-B-type natriuretic peptide (NTproBNP), and troponin-T compared to those untreated (all *p* < 0.05). In contrast, baseline measurements of left ventricular ejection fraction (LVEF), left atrial volume (LAV), and right ventricular ejection fraction (RVEF) showed no significant differences based on the tafamidis treatment status (all *p* > 0.05).

**Figure 1:**
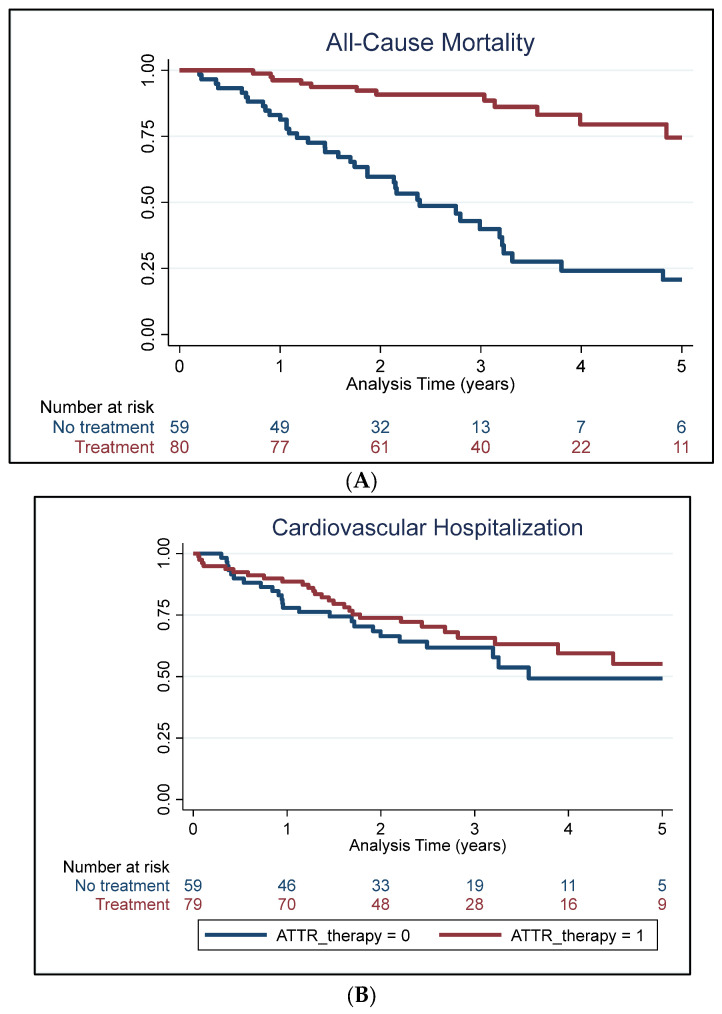

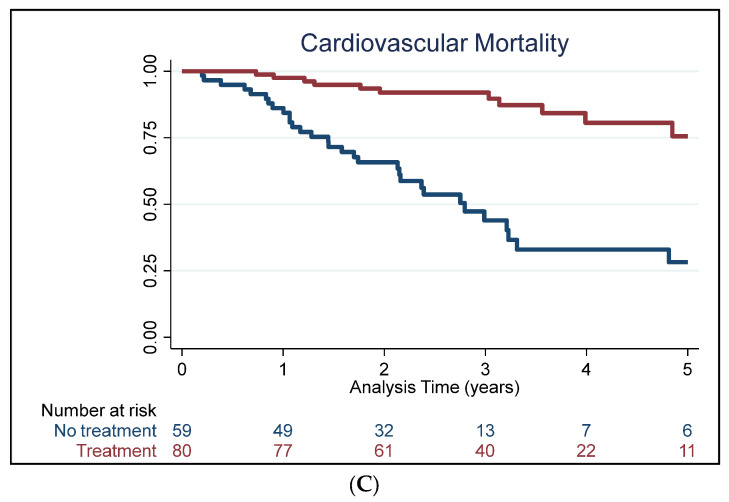
Kaplan–Meier survival curves for all-cause mortality (**A**), cardiovascular mortality (**B**), and hospitalization (**C**) stratified by tafamidis therapy.

The mean follow-up was 2.9 ± 1.8 years. Over the duration of the study, there were a total of 57 deaths, of which 49 were cardiovascular-related (Table 2). Patients in the tafamidis-treated group were less likely to experience all-cause and cardiovascular mortality overall compared to those untreated (both *p* < 0.05). Mortality exceeded 50% among the untreated patients. The number of cardiovascular hospitalizations experienced in the cohort did not significantly differ by treatment status. Notably, the median survival time for experiencing all-cause and cardiovascular mortality in patients not on therapy was 2.4 and 2.8 years, respectively, whereas those receiving treatment had a median survival time of 8.2 years (Figure 1). Supplementary Figure S1 presents baseline and 1-year follow-up blood pressure values for patients treated and untreated with tafamidis.

### 5.2. Univariable Outcomes Analysis

Variables associated with all-cause and cardiovascular mortality are described in Table 3. The only baseline variable associated with cardiovascular hospitalization was the estimated glomerular filtration rate (eGFR). Baseline eGFR demonstrated a significant interaction with tafamidis, with lower values of eGFR at the baseline associated with less cardiovascular hospitalization benefit from treatment (interaction HR [95% CI], 0.89 [0.81–0.99], *p* < 0.05).

Patients receiving tafamidis therapy exhibited a 57% and 52% reduction in the risk of all-cause and cardiovascular death, respectively, compared to those untreated (unadjusted HR [95% CI], all-cause mortality 0.43 [0.23–0.80] and cardiovascular mortality 0.48 [0.25–0.93], both *p* < 0.05; Table 3). No significant differences were observed for cardiovascular hospitalization when comparing individuals on and not on therapy (unadjusted HR [95% CI], 0.84 [0.44–1.60], *p* > 0.05).

A higher NTproBNP value at baseline was associated with increased all-cause (HR per 1000 ng/L [95% CI], 1.07 [1.04–1.10], *p* < 0.05) and cardiovascular mortality (HR per 1000 ng/L [95% CI], 1.08 [1.05–1.11], *p* < 0.05). There was also a significant interaction with tafamidis such that a higher NTproBNP was associated with less benefit from therapy (interaction HR [95% CI], all-cause mortality 1.09 [1.01–1.20], cardiovascular mortality 1.08 [1.01–1.15], and both *p* < 0.05). Lower eGFR at baseline was associated with increased all-cause (HR [95% CI], 0.89 [0.81–0.97], *p* < 0.05) and cardiovascular mortality (HR [95% CI], 0.89 [0.84–0.95], *p* < 0.05). There was also a significant interaction with tafamidis, such that a lower eGFR was associated with less benefit from therapy (HR [95% CI], all-cause mortality 0.09 [0.08–0.09], and cardiovascular mortality 0.004 [0.003–0.004], *p* < 0.05).

Multiple imaging parameters were individually significantly associated with all-cause mortality, with a smaller number associated with cardiovascular mortality (Table 3). Measures of left and right ventricular function were found to be significant individual outcome predictors for all-cause mortality. Supplementary Tables S1 and S2 present unadjusted HRs for patients treated and not treated with tafamidis for study outcomes, respectively.

### 5.3. Multivariable Outcome Analysis

Clinical, biochemical, and cardiac imaging variables significantly associated with all-cause and cardiovascular mortality after the multivariable analysis are described in Table 4. Frailty (clinical frailty score ≥ 4) demonstrated a significant association with both all-cause and cardiovascular mortality (HR [95% CI], all-cause mortality 12.84 [2.58–63.86], cardiovascular mortality 8.87 [2.67–29.42], both *p* < 0.05), as did age. eGFR was the only variable associated with cardiovascular hospitalization and did not interact with treatment in the model. Poor renal function was also a significant predictor of all-cause and cardiovascular mortality (both *p* < 0.05) in the multivariable model.

Left atrial volume (LAV) was significantly associated with cardiovascular mortality (HR [95% CI], 1.02 [1.01–1.04], *p* < 0.001), but not all-cause mortality. No left ventricular parameters from the univariable analysis were significant in multivariable modeling for both all-cause and cardiovascular mortality. However, measures of right ventricular function were found to be significant predictors of both all-cause and cardiovascular mortality (Table 4). Notably, there were no variables that significantly interacted with tafamidis treatment for all-cause and cardiovascular mortality in the multivariable model. Supplementary Tables S3 and S4 present adjusted HRs for patients treated and not treated with tafamidis for the study outcomes, respectively.

## 6. Discussion

Our study investigated associations of baseline parameters across clinical, biochemical, and cardiac imaging domains to understand their impact on major adverse events, including all-cause mortality, cardiovascular mortality, and hospitalization in ATTR-CM patients followed at a Canadian referral center. Additionally, we explored whether the baseline parameters significantly associated with outcome modify the benefit of tafamidis treatment. The primary findings of this study reveal significant associations of frailty, blood pressure, kidney function, and RV structure and function with all-cause mortality. Importantly, these factors did not interact with the use of tafamidis therapy in the multivariable analysis, suggesting their prognostic significance irrespective of treatment status. These same factors, along with left atrial volume, were all associated with cardiovascular mortality, while kidney function was the only factor linked to cardiovascular hospitalization in this study.

The ATTR-ACT trial demonstrated a significant survival benefit associated with tafamidis therapy over the placebo for ATTR-CM patients [5]. Exclusion criteria included the following: age >90 years, NYHA functional class IV symptoms, eGFR < 25 mL/min/1.73 m^2^, liver transaminases above two times the upper limit of normal, and a severely reduced modified body mass index [5]. While criteria for public reimbursement in Canada vary somewhat across provinces (and other countries), generally, they are similar and follow the Canadian Agency for Drug and Technology in Health (CADTH) recommendations, which only include NYHA functional class IV symptoms as an exclusion to eligibility among the criteria listed above and do not include age [6,12]. This can make identifying patients less likely to benefit from therapy challenging in clinical practice. This is particularly important when considering both the high cost of tafamidis and that a survival benefit emerged after 18 months of treatment in the ATTR-ACT trial [5,13]. These characteristics make improved understanding of factors associated with outcome in clinical practice of critical importance in this predominantly older patient population.

Several studies have explored the use of tafamidis in real-world populations of ATTR-CM patients, demonstrating that adverse outcome-free survival is achievable with this therapy, regardless of disease subtype [14]. Patients initially randomized to placebo in the ATTR-ACT trial had poorer disease prognoses over time, as observed in the long-term extension study, compared to those initially randomized to tafamidis [15,16]. Although survival stabilized in both groups, these findings underscore the importance of early diagnosis and treatment.

A significant interaction with treatment was observed for NTproBNP in our univariable analysis, suggesting that patients with worsening heart failure were less likely to benefit from therapy compared to others. Higher eGFR also interacted with treatment in the univariable analysis, indicating that patients with better kidney function were more likely to benefit from therapy compared to those with poor kidney function. Neither variable interacted with tafamidis treatment in multivariable modeling.

Surprisingly, the multivariable modeling results demonstrated no LV structural or functional parameters (such as EF, end-diastolic volume (EDV), ESV) or cardiac biomarkers (NTproBNP and troponin-T) that were significantly associated with all-cause and cardiovascular mortality. This suggests that, clinically, in patients with ATTR-CM, RV function may be a more valuable marker for assessing the risk of all-cause and cardiovascular mortality than LV function. Previous studies have also demonstrated the prognostic significance of RV dysfunction in ATTR-CM [17,18].

## 7. Limitations

This study had a single-center retrospective observational design; therefore, the presence of bias cannot be excluded. Patients taking and not taking tafamidis were compared, however not randomly assigned, further introducing potential bias into the analysis despite reflecting clinical (‘real-world’) decision-making practice. The study period includes patients diagnosed with ATTR-CM before and after the approval of clinical availability of tafamidis in Canada (2020), and therefore, tafamidis use was analyzed via regression modeling as a time-varying covariate; however, this may not entirely correct for potential bias.

## 8. Conclusions

This study provides insights into the determinants of prognosis for patients with ATTR-CM, elucidating factors associated with adverse outcomes from a Canadian referral center. Important baseline parameters influencing adverse outcomes in ATTR-CM patients included frailty, blood pressure, kidney function, and RV function, all contributing significantly to all-cause mortality. These factors did not interact with the use of tafamidis therapy in multivariable analysis, suggesting their prognostic significance irrespective of treatment status. Furthermore, these identified factors, in addition to left atrial volume, were associated with cardiovascular mortality, while the only factor associated with cardiovascular hospitalization was renal function. These findings may be of value for updating criteria for improving selection of ATTR-CM patients eligible for tafamidis who are most likely to derive benefit.

## Figures and Tables

**Table 1:** Baseline patient characteristics. Variables are described for all patients, and for those treated and untreated with tafamidis. Asterisk (*) represents missing data for some patients.

Characteristics	All Patients (*n* = 139)	Tafamidis (*n* = 80)	Not on Tafamidis (*n* = 59)	*p*-Value
Clinical				
Age at diagnosis (years), median (IQR)	80.9 (74.3–86.6)	77.8 (71.3–83.3)	86.3 (79.1–88.8)	<0.001
Sex male, *n* (%)	121 (87.1)	70 (87.5)	51 (86.4)	1.000
hATTR, *n* (%)	8 (5.8)	7 (8.8)	1 (1.7)	0.138
BMI (kg/m^2^), median (IQR)	26.6 (23.9–29.7)	26.6 (24.0–30.7)	26.3 (23.6–28.8)	0.441
Comorbidities, *n* (%)				
Atrial fibrillation or flutter	101 (72.7)	55 (68.8)	46 (78.0)	0.253
Aortic stenosis *	22 (17.1)	11 (14.1)	11 (21.6)	0.339
Pacemaker or ICD	40 (28.8)	21 (26.3)	19 (32.2)	0.455
Hypertension	90 (64.8)	45 (56.3)	45 (76.3)	0.019
Diabetes	27 (19.4)	13 (16.3)	14 (23.7)	0.286
Carpal tunnel syndrome	99 (71.2)	69 (86.3)	30 (50.9)	<0.001
Polyneuropathy	48 (34.5)	33 (41.3)	15 (25.4)	0.071
Spinal stenosis	37 (26.6)	22 (27.5)	15 (25.4)	0.847
Amyloidosis Scores, *n* (%)				
PND score ≥ IIIa	50 (36.0)	21 (26.3)	29 (49.2)	0.007
Clinical frailty scale ≥ 4	103 (74.1)	49 (61.3)	54 (91.5)	<0.001
Concomitant Medications, *n* (%)				
Anticoagulants	106 (76.3)	60 (75.0)	46 (78.0)	0.840
Diuretics	119 (85.6)	69 (86.3)	50 (84.8)	0.812
Biochemical, median (IQR)				
Creatinine (µmol/L) *	103.0 (87.0–128.0)	96.0 (84.0–121.0)	117.0 (93.0–149.0)	0.002
NTproBNP (ng/L) *	3591.0 (1779.0–6451.0)	3091.0 (1471.0–4305.0)	6179.5 (2913.0–10,872.0)	0.001
Troponin-T (ng/L) *	65.0 (42.0–104.0)	55.0 (35.0–74.0)	86.0 (56.0–132.0)	0.005
Cardiac Imaging				
ECHO LVEF (%), mean (SD) *	46.8 ± 11.9	47.2 ± 11.9	46.3 ± 12.2	0.721
CMR RVEF (%), mean (SD) *	48.1 ± 11.8	47.9 ± 12.2	49.1 ± 10.7	0.708
ECHO LAV (mL), median (IQR) *	89.0 (75.4–110.0)	94.0 (74.9–112.1)	87.7 (75.6–107.1)	0.679

**Table 2:** Major adverse outcomes of the study population. Variables are described for all patients, and for those treated and not treated with tafamidis therapy.

Outcomes, *n* (%)	All Patients (*n* = 139)	Tafamidis (*n* = 80)	Not on Tafamidis (*n* = 59)	*p*-Value
All-cause mortality	57 (41.0)	15 (18.8)	42 (71.2)	<0.001
CV mortality	49 (35.3)	13 (16.3)	36 (61.0)	<0.001
CV hospitalization	54 (38.9)	29 (36.3)	25 (42.4)	0.486

**Table 3:** Unadjusted hazard ratios from univariable survival analysis. Asterisk (*) represents significant interaction with tafamidis treatment (*p* < 0.05). No median imputation was performed.

	All-Cause Mortality		Cardiovascular Mortality		Cardiovascular Hospitalization	
Characteristics	HR (95% CI)	*p*-Value	HR (95% CI)	*p*-Value	HR (95% CI)	*p*-Value
Tafamidis	0.43 (0.23–0.80)	0.008	0.48 (0.25–0.93)	0.031	0.84 (0.44–1.60)	0.596
Clinical						
Age at diagnosis	1.13 (1.08–1.18)	<0.001	1.11 (1.07–1.16)	<0.001	1.02 (0.98–1.06)	0.299
Male	0.99 (0.44–2.22)	0.972	0.97 (0.36–2.64)	0.954	0.87 (0.38–1.96)	0.729
Atrial fibrillation	2.90 (1.31–6.41)	0.008	4.69 (1.61–13.69)	0.005	1.61 (0.84–3.09)	0.155
NYHA functional class ≥ III	2.22 (1.30–3.80)	0.004	2.44 (1.41–4.21)	0.001	1.41 (0.82–2.40)	0.213
PND score ≥ IIIa	2.46 (1.46–4.16)	0.001	2.15 (1.24–3.75)	0.007	1.05 (0.61–1.80)	0.863
Clinical frailty scale ≥ 4	13.92 (3.39–57.20)	<0.001	10.63 (2.71–41.66)	0.001	1.57 (0.81–3.04)	0.184
Anticoagulants	2.15 (0.97–4.75)	0.060	2.67 (1.01–7.04)	0.047	1.12 (0.57–2.21)	0.737
Systolic BP per 10	0.84 (0.71–0.99)	0.041	0.80 (0.66–0.97)	0.023	1.00 (0.87–1.15)	0.996 *
Diastolic BP per 10	0.63 (0.46–0.88)	0.006	0.58 (0.41–0.82)	0.002	0.83 (0.61–1.12)	0.221
Biochemical						
NTproBNP per 1000 ng/L	1.07 (1.04–1.10)	<0.001 *	1.08 (1.05–1.11)	<0.001 *	0.99 (0.95–1.03)	0.583
Troponin T per 100 ng/L	1.39 (1.18–1.63)	<0.001	1.43 (1.26–1.63)	<0.001	0.98 (0.80–1.22)	0.883
Albumin	0.91 (0.85–0.98)	0.009	0.91 (0.85–0.97)	0.004	0.98 (0.92–1.05)	0.585
Hemoglobin	0.97 (0.95–0.99)	<0.001	0.98 (0.96–0.99)	0.026	1.00 (0.99–1.01)	0.995
Platelets per 10	0.96 (0.92–1.00)	0.087	0.96 (0.90–1.02)	0.171	1.00 (0.96–1.04)	0.991 *
Creatinine per 10	1.09 (1.05–1.14)	<0.001	1.08 (1.03–1.13)	0.002	1.01 (0.95–1.06)	0.814
Na	0.88 (0.81–0.95)	0.002	0.87 (0.80–0.95)	0.003	0.95 (0.87–1.05)	0.323
eGFR	0.89 (0.81–0.97)	0.011 *	0.89 (0.84–0.95)	<0.001 *	0.94 (0.90–0.98)	0.006 *
Imaging						
CMR RVEDV	1.01 (1.00–1.01)	0.011 *	1.00 (0.99–1.01)	0.867	1.00 (1.00–1.01)	0.732
CMR RVEDV_i	1.02 (1.00–1.03)	0.024 *	0.99 (0.97–1.02)	0.612	1.00 (0.99–1.01)	0.995
CMR RVESV	1.01 (1.00–1.02)	0.004 *	1.00 (0.99–1.01)	0.683 *	1.00 (0.99–1.01)	0.906
CMR RVESV_i	1.02 (1.01–1.03)	0.006 *	1.00 (0.98–1.02)	0.875 *	1.00 (0.98–1.01)	0.682
CMR RVEF	0.97 (0.94–1.00)	0.065	0.98 (0.95–1.02)	0.319 *	1.01 (0.99–1.04)	0.388
CMR LAV	1.02 (1.01–1.03)	0.001	1.02 (1.01–1.03)	0.005	1.00 (0.99–1.01)	0.922
CMR LAV_i	1.04 (1.01–1.06)	0.007	1.04 (1.01–1.07)	0.021	1.01 (0.99–1.03)	0.328
ECHO LVEDV	0.98 (0.97–0.99)	0.029 *	0.99 (0.97–1.00)	0.146	1.01 (1.00–1.03)	0.079 *
ECHO LVEF	0.97 (0.94–0.99)	0.039	0.98 (0.95–1.01)	0.139	0.99 (0.97–1.02)	0.671
ECHO LAV	1.01 (1.00–1.03)	0.010	1.02 (1.01–1.03)	<0.001	1.00 (0.99–1.01)	0.410
ECHO LAV_i	1.04 (1.02–1.06)	<0.001	1.05 (1.03–1.07)	<0.001	1.01 (0.99–1.03)	0.315

**Table 4:** Adjusted hazard ratios from the multivariable model after performing median imputation for selected continuous variables with missing observations.

	All-Cause Mortality	
Characteristics	HR (95% CI)	*p*-Value
Clinical		
Age at diagnosis	1.15 (1.09–1.22)	<0.001
Clinical frailty scale ≥ 4	12.84 (2.58–63.86)	0.002
Systolic BP per 10	0.68 (0.53–0.86)	0.002
Biochemical		
Creatinine per 10	1.06 (1.01–1.12)	0.022
Na	0.89 (0.81–0.97)	0.006
Imaging		
CMR RVESV_i	1.12 (1.01–1.24)	0.033
CMR RVEF	1.14 (1.02–1.26)	0.017
	Cardiovascular Mortality	
Characteristics	HR (95% CI)	*p*-value
Clinical		
Age at diagnosis	1.12 (1.07–1.17)	<0.001
Clinical frailty scale ≥ 4	8.87 (2.67–29.42)	<0.001
Systolic BP per 10	0.68 (0.53–0.87)	0.002
Biochemical		
Creatinine per 10	1.07 (1.02–1.11)	0.005
Imaging		
CMR RVEDV_i	0.94 (0.90–0.98)	0.003
CMR RVESV	1.03 (1.00–1.06)	0.022
CMR RVEF	1.07 (1.02–1.14)	0.013
ECHO LAV	1.02 (1.01–1.04)	<0.001

## Data Availability

Data from this study are available upon reasonable request to the corresponding author.

## Supplementary Materials

The following supporting information can be downloaded at https://www.mdpi.com/article/10.3390/jcm13185490/s1. Figure S1: Baseline and 1-year follow-up systolic and diastolic blood pressure for patients treated and untreated with tafamidis disease-modifying therapy. Observations: baseline (*n* = 67 for tafamidis, *n* = 41 for non-tafamidis) and 1-year follow-up (*n* = 51 for tafamidis, *n* = 18 for non-tafamidis); Table S1: Unadjusted hazard ratios from univariable survival analysis for tafamidis-treated patients. No median imputation performed; Table S2: Unadjusted hazard ratios from univariable survival analysis for untreated patients. No median imputation performed; Table S3: Adjusted hazard ratios from multivariable model after performing median imputation for selected continuous variables with miss-ing observations for tafamidis-treated patients; and Table S4: Adjusted hazard ratios from multivariable model after performing median imputation for selected continuous variables with miss-ing observations for untreated patients.

## Abbreviations

ATTR-ACT	The Transthyretin Amyloidosis Cardiomyopathy Clinical Trial
ATTR-CM	transthyretin amyloidosis cardiomyopathy
BMI	body mass index
CI	confidence interval
CIROC	Cardiovascular Imaging Registry of Calgary
CMR	cardiovascular magnetic resonance
ECG	electrocardiogram
ECHO	echocardiography
EDV	end-diastolic volume
EF	ejection fraction
ESV	end-systolic volume
eGFR	estimated glomerular filtration rate
hATTR	hereditary ATTR-CM disease subtype
HF	heart failure
ICD	implantable cardioverter-defibrillator
ICD-10	international classification of diseases, tenth revision
IQR	interquartile range
LAV	left atrial volume
LV	left ventricle
NTproBNP	N-terminal pro-B-type natriuretic peptide
NYHA	New York Heart Association
PND	polyneuropathy disability
RV	right ventricle
Tc-99m-PYP	Technetium-99m Pyrophosphate
TTR	Transthyretin/Pre-Albumin Protein
wtATTR	wild-type ATTR-CM disease subtype

## References

[1] Donnelly J.P., Hanna M. (2017). Cardiac amyloidosis: An update on diagnosis and treatment. Clevel. Clin. J. Med..

[2] Fine N.M., Davis M.K., Anderson K., Delgado D.H., Giraldeau G., Kitchlu A., Massie R., Narayan J., Swiggum E., Venner C.P. (2020). Canadian Cardiovascular Society/Canadian Heart Failure Society Joint Position Statement on the Evaluation and Management of Patients with Cardiac Amyloidosis. Can. J. Cardiol..

[3] AbouEzzeddine O.F., Davies D.R., Scott C.G., Fayyaz A.U., Askew J.W., McKie P.M., Noseworthy P.A., Johnson G.B., Dunlay S.M., Borlaug B.A. (2021). Prevalence of Transthyretin Amyloid Cardiomyopathy in Heart Failure with Preserved Ejection Fraction. JAMA Cardiol..

[4] Ruberg F.L., Grogan M., Hanna M., Kelly J.W., Maurer M.S. (2019). Transthyretin Amyloid Cardiomyopathy. J. Am. Coll. Cardiol..

[5] Maurer M.S., Schwartz J.H., Gundapaneni B., Elliott P.M., Merlini G., Waddington-Cruz M., Kristen A.V., Grogan M., Witteles R., Damy T. (2018). Tafamidis Treatment for Patients with Transthyretin Amyloid Cardiomyopathy. New Engl. J. Med..

[6] (2020). Pharmacoeconomic Review Report: Tafamidis (Vyndaqel): (Pfizer Canada ULC): Indication: For the Treatment of Adult Patients with Cardiomyopathy Due to Transthyretin-Mediated Amyloidosis, Wild-Type or Hereditary, to Reduce Cardiovascular Mortality and Cardiovascular-Related Hospitalization.

[7] Garcia-Pavia P., Rapezzi C., Adler Y., Arad M., Basso C., Brucato A., Burazor I., Caforio A.L., Damy T., Eriksson U. (2021). Diagnosis and treatment of cardiac amyloidosis: A position statement of the ESC Working Group on Myocardial and Pericardial Diseases. Eur. Heart J..

[8] Cornhill A.K., Dykstra S., Satriano A., Labib D., Mikami Y., Flewitt J., Prosio E., Rivest S., Sandonato R., Howarth A.G. (2022). Machine Learning Patient-Specific Prediction of Heart Failure Hospitalization Using Cardiac MRI-Based Phenotype and Electronic Health Information. Front. Cardiovasc. Med..

[9] Yamamoto S., Wilczek H.E., Nowak G., Larsson M., Oksanen A., Iwata T., Gjertsen H., Söderdahl G., Wikström L., Ando Y. (2007). Liver transplantation for familial amyloidotic polyneuropathy (FAP): A single-center experience over 16 years. Am. J. Transplant..

[10] Rockwood K., Theou O. (2020). Using the clinical frailty scale in allocating scarce health care resources. Can. Geriatr. J..

[11] Rios R., Miller R.J.H., Manral N., Sharir T., Einstein A.J., Fish M.B., Ruddy T.D., Kaufmann P.A., Sinusas A.J., Miller E.J. (2022). Handling missing values in machine learning to predict patient-specific risk of adverse cardiac events: Insights from REFINE SPECT registry. Comput. Biol. Med..

[12] (2020). CADTH Common Drug Review: Canadian Drug Expert Reimbursement Recommendations: Tafamidis Meglumine.

[13] Garcia-Pavia P., Bengel F., Brito D., Damy T., Duca F., Dorbala S., Nativi-Nicolau J., Obici L., Rapezzi C., Sekijima Y. (2021). Expert consensus on the monitoring of transthyretin amyloid cardiomyopathy. Eur. J. Heart Fail..

[14] Bézard M., Kharoubi M., Galat A., Poullot E., Guendouz S., Fanen P., Funalot B., Moktefi A., Lefaucheur J.P., Abulizi M. (2021). Natural history and impact of treatment with tafamidis on major cardiovascular outcome-free survival time in a cohort of patients with transthyretin amyloidosis. Eur. J. Heart Fail..

[15] Damy T., Garcia-Pavia P., Hanna M., Judge D.P., Merlini G., Gundapaneni B., Patterson T.A., Riley S., Schwartz J.H., Sultan M.B. (2021). Efficacy and safety of tafamidis doses in the Tafamidis in Transthyretin Cardiomyopathy Clinical Trial (ATTR-ACT) and long-term extension study. Eur. J. Heart Fail..

[16] Elliott P., Drachman B.M., Gottlieb S.S., Hoffman J.E., Hummel S.L., Lenihan D.J., Ebede B., Gundapaneni B., Li B., Sultan M.B. (2022). Long-term survival with tafamidis in patients with transthyretin amyloid cardiomyopathy. Circ. Heart Fail..

[17] Bodez D., Ternacle J., Guellich A., Galat A., Lim P., Radu C., Guendouz S., Bergoend E., Couetil J.P., Hittinger L. (2016). Prognostic value of right ventricular systolic function in cardiac amyloidosis. Amyloid.

[18] Arvidsson S., Henein M.Y., Wikström G., Suhr O.B., Lindqvist P. (2018). Right ventricular involvement in transthyretin amyloidosis. Amyloid.

