# Supplementary material for: Baseline Predictors of Adverse Outcomes for Transthyretin Amyloidosis Cardiomyopathy Patients Treated and Untreated with Tafamidis: A Canadian Referral Center Experience
Source: J Clin Med. 2024 Sep 16;13(18):5490. doi: 10.3390/jcm13185490 (PMC11432679; doi:10.3390/jcm13185490)

Supplementary Materials

Table S1. Unadjusted hazard ratios from univariable survival analysis for tafamidis-treated patients. No median imputation performed.

	**All-cause mortality**		**Cardiovascular mortality**		**Cardiovascular hospitalization**	
**Characteristics**	**HR (95% CI)**	**P-value**	**HR (95% CI)**	**P-value**	**HR (95% CI)**	**P-value**
Clinical						
Age at diagnosis	1.14 (1.04-1.24)	**0.003**	1.14 (1.05-1.24)	**0.002**	1.06 (0.96-1.18)	0.267
Male	2.93 (0.36-23.50)	0.313	2.25 (0.22-23.21)	0.495	0.85 (0.18-4.13)	0.844
Atrial Fibrillation	3.79 (0.85-16.97)	0.082	3.00 (0.67-13.41)	0.151	1.44 (0.46-4.54)	0.533
NYHA Functional Class ≥III	0.90 (0.24-3.42)	0.875	1.09 (0.31-3.88)	0.889	1.21 (0.37-3.93)	0.756
PND Score ≥ IIIa	2.18 (0.69-6.87)	0.181	2.62 (0.89-7.72)	0.080	0.93 (0.25-3.44)	0.908
Clinical Frailty Scale ≥ 4	16.14 (2.09-124.75)	**0.008**	12.79 (1.76-92.73)	**0.012**	1.40 (0.50-3.92)	0.525
Anticoagulants	2.59 (0.58-11.64)	0.215	2.15 (0.46-9.97)	0.327	1.51 (0.42-5.40)	0.529
Systolic BP per 10	0.92 (0.69-1.23)	0.577	0.96 (0.72-1.30)	0.808	1.27 (0.96-1.67)	0.099
Diastolic BP per 10	0.72 (0.42-1.23)	0.229	0.77 (0.47-1.27)	0.304	1.06 (0.67-1.68)	0.809
Biochemical						
NTproBNP per 1000 ng/L	1.18 (1.07-1.30)	**0.001**	1.18 (1.12-1.25)	**<0.001**	0.99 (0.92-1.07)	0.819
Troponin T per 100 ng/L	1.77 (1.05-3.01)	**0.034**	1.77 (1.20-2.62)	**0.004**	0.85 (0.59-1.24)	0.406
Albumin	0.92 (0.79-1.07)	0.284	0.87 (0.75-1.01)	0.073	0.95 (0.85-1.05)	0.304
Hemoglobin	0.97 (0.93-1.01)	0.096	0.99 (0.96-1.03)	0.651	0.99 (0.96-1.03)	0.799
Platelets per 10	0.97 (0.88-1.07)	0.577	0.99 (0.89-1.10)	0.820	0.91 (0.84-0.98)	**0.013**
Creatinine per 10	1.05 (0.94-1.17)	0.379	1.05 (0.94-1.17)	0.412	1.07 (0.99-1.16)	0.066
Na	0.92 (0.79-1.08)	0.312	0.89 (0.73-1.10)	0.287	1.00 (0.85-1.18)	0.970
eGFR	0.22	**.**	0.15 (0.14-0.15)	**<0.001**	0.0000336	**.**
Imaging						
CMR RVEDV	1.02 (1.01-1.03)	**0.002**	1.01 (1.00-1.02)	0.201	1.00 (0.99-1.01)	0.710
CMR RVEDV_i	1.04 (1.02-1.07)	**0.002**	1.02 (0.99-1.04)	0.201	1.00 (0.98-1.02)	0.711
CMR RVESV	1.02 (1.01-1.03)	**0.001**	1.01 (1.00-1.02)	**0.044**	1.00 (0.99-1.01)	0.722
CMR RVESV_i	1.05 (1.02-1.08)	**0.001**	1.03 (1.00-1.06)	**0.031**	1.00 (0.97-1.02)	0.724
CMR RVEF	0.94 (0.90-0.99)	**0.030**	0.93 (0.87-0.99)	**0.040**	1.00 (0.97-1.04)	0.864
CMR LAV	1.02 (1.01-1.04)	**0.007**	1.02 (1.01-1.04)	**0.005**	1.01 (0.99-1.02)	0.197
CMR LAV_i	1.04 (1.00-1.08)	**0.033**	1.05 (1.01-1.09)	**0.013**	1.02 (0.99-1.04)	0.124
ECHO LVEDV	1.00 (0.98-1.02)	0.939	1.00 (0.98-1.02)	0.926	1.01 (0.97-1.02)	0.634
ECHO LVEF	0.96 (0.92-1.01)	0.149	0.96 (0.90-1.03)	0.246	1.01 (0.96-1.06)	0.677
ECHO LAV	1.03 (1.01-1.05)	**0.007**	1.02 (1.01-1.05)	**0.005**	1.00 (0.99-1.01)	0.940
ECHO LAV_i	1.07 (1.04-1.11)	**<0.001**	1.07 (1.04-1.10)	**<0.001**	1.01 (0.99-1.04)	0.310

Table S2. Unadjusted hazard ratios from univariable survival analysis for untreated patients. No median imputation performed.

	All-cause mortality		Cardiovascular mortality		Cardiovascular hospitalization	
**Characteristics**	**HR (95% CI)**	**P-value**	**HR (95% CI)**	**P-value**	**HR (95% CI)**	**P-value**
Clinical						
Age at diagnosis	1.13 (1.07-1.19)	**<0.001**	1.10 (1.04-1.15)	**<0.001**	1.01 (0.97-1.05)	0.721
Male	0.48 (0.20-1.16)	0.101	0.54 (0.20-1.50)	0.238	0.83 (0.33-2.07)	0.690
Atrial Fibrillation	2.02 (0.79-5.17)	0.143	5.39 (1.23-23.65)	**0.026**	1.64 (0.73-3.68)	0.227
NYHA Functional Class ≥III	2.57 (1.38-4.76)	**0.003**	2.90 (1.54-5.44)	**0.001**	1.53 (0.83-2.81)	0.170
PND Score ≥ IIIa	2.66 (1.39-5.10)	**0.003**	1.82 (0.97-3.42)	0.062	1.06 (0.58-1.95)	0.839
Clinical Frailty Scale ≥ 4	9.08 (1.24-66.32)	**0.030**	7.27 (1.15- 45.86)	**0.035**	1.82 (0.70-4.75)	0.221
Anticoagulants	1.45 (0.56-3.75)	0.440	2.53 (0.75-8.59)	0.136	0.98 (0.44-2.16)	0.953
Systolic BP per 10	0.80 (0.63-1.01)	0.055	0.67 (0.53-0.86)	**0.001**	0.88 (0.75-1.04)	0.122
Diastolic BP per 10	0.69 (0.44-1.08)	0.103	0.51 (0.33-0.77)	**0.001**	0.71 (0.48-1.05)	0.085
Biochemical						
NTproBNP per 1000 ng/L	1.05 (1.01-1.09)	**0.006**	1.06 (1.03-1.09)	**<0.001**	0.99 (0.94-1.04)	0.668
Troponin T per 100 ng/L	1.28 (1.01-1.64)	**0.045**	1.30 (1.07-1.58)	**0.007**	1.19 (0.94-1.50)	0.156
Albumin	0.93 (0.85-1.01)	0.076	0.92 (0.85-1.00)	0.062	0.99 (0.92-1.07)	0.842
Hemoglobin	0.97 (0.95-0.99)	**0.001**	0.97 (0.95-0.99)	**0.011**	1.00 (0.98-1.02)	0.930
Platelets per 10	0.96 (0.91-1.01)	0.091	0.95 (0.88-1.02)	0.135	1.02 (0.98-1.05)	0.361
Creatinine per 10	1.10 (1.04-1.16)	**<0.001**	1.09 (1.03-1.14)	**0.001**	0.98 (0.91-1.06)	0.614
Na	0.83 (0.75-0.92)	**<0.001**	0.84 (0.76-0.93)	**0.001**	0.93 (0.84-1.04)	0.205
eGFR	0.91 (0.84-0.99)	**0.040**	0.91 (0.87-0.96)	**<0.001**	0.95 (0.91-0.99)	**0.035**
Imaging						
CMR RVEDV	1.00 (0.99-1.01)	0.717	0.99 (0.98-1.01)	0.278	1.00 (0.99-1.01)	0.390
CMR RVEDV_i	1.00 (0.98-1.02)	0.892	0.97 (0.93-1.01)	0.115	1.00 (0.99-1.01)	0.735
CMR RVESV	1.00 (0.99-1.01)	0.636	0.99 (0.98-1.00)	0.206	1.00 (0.99-1.01)	0.751
CMR RVESV_i	1.00 (0.98-1.03)	0.699	0.98 (0.94-1.01)	0.125	1.00 (0.99-1.01)	0.945
CMR RVEF	1.00 (0.95-1.04)	0.863	1.02 (0.97-1.06)	0.463	1.01 (0.98-1.04)	0.436
CMR LAV	1.02 (1.00-1.04)	0.093	1.01 (0.99-1.03)	0.225	0.99 (0.98-1.01)	0.379
CMR LAV_i	1.04 (0.99-1.08)	0.119	1.02 (0.97-1.07)	0.363	1.00 (0.97-1.04)	0.844
ECHO LVEDV	0.97 (0.95-0.99)	**0.003**	0.98 (0.95-0.99)	**0.023**	1.02 (1.00-1.03)	**0.015**
ECHO LVEF	0.99 (0.95-1.02)	0.450	0.99 (0.96-1.03)	0.686	0.99 (0.96-1.02)	0.389
ECHO LAV	1.01 (0.99-1.03)	0.207	1.02 (1.00-1.04)	**0.013**	1.01 (0.99-1.02)	0.167
ECHO LAV_i	1.02 (0.99-1.05)	0.154	1.04 (1.01-1.07)	**0.013**	1.01 (0.98-1.03)	0.610

Table S3. Adjusted hazard ratios from multivariable model after performing median imputation for selected continuous variables with missing observations for tafamidis-treated patients.

	**All-cause mortality**	
**Characteristics**	**HR (95% CI)**	**P-value**
Clinical		
Age at diagnosis	1.17 (1.04-1.32)	**0.008**
Clinical Frailty Scale ≥ 4	26.31 (2.29-302.71)	**0.009**
Imaging		
CMR RVEDV_i	0.87 (0.76-0.99)	**0.048**
CMR RVESV_i	1.33 (1.06-1.67)	**0.014**
CMR RVEF	1.32 (1.05-1.67)	**0.018**

	**Cardiovascular Mortality**	
**Characteristics**	**HR (95% CI)**	**P-value**
Clinical		
Age at diagnosis	1.23 (1.10-1.37)	**<0.001**
Male	7.86 (1.03-59.96)	**0.047**
Atrial Fibrillation	2.52×10^{9} (4.51×10^{7}-1.41×10^{11})	**<0.001**
Anticoagulants	8.60×10^{-10} (1.49×10^{-11}-4.96×10^{-8})	**<0.001**
Biochemical		
eGFR	0.90 (0.82-0.99)	**0.026**
Imaging		
CMR RVEDV_i	0.74 (0.61-0.89)	**0.002**
CMR RVESV_i	1.58 (1.19-2.10)	**0.002**
CMR RVEF	1.36 (1.09-1.70)	**0.007**

	**Cardiovascular Hospitalization**	
**Characteristics**	**HR (95% CI)**	**P-value**
Biochemical		
eGFR	0.89 (0.83-0.96)	**0.002**

Table S4. Adjusted hazard ratios from multivariable model after performing median imputation for selected continuous variables with missing observations for untreated patients.

	All-cause mortality	
**Characteristics**	**HR (95% CI)**	**P-value**
Clinical		
Age at diagnosis	1.15 (1.08-1.22)	**<0.001**
Male	0.39 (0.16-0.99)	**0.049**
PND Score ≥ IIIa	2.75 (1.40-5.43)	**0.003**
Systolic BP per 10	0.62 (0.46-0.83)	**0.001**
Biochemical		
eGFR	0.95 (0.92-0.99)	**0.013**

	Cardiovascular Mortality	
**Characteristics**	**HR (95% CI)**	**P-value**
Clinical		
Age at diagnosis	1.13 (1.07-1.19)	**<0.001**
NYHA Functional Class ≥III	2.30 (1.28-4.13)	**0.005**
Systolic BP per 10	0.57 (0.45-0.74)	**<0.001**
Biochemical		
eGFR	0.96 (0.93-0.99)	**0.010**
Imaging		
CMR RVESV_i	0.96 (0.94-0.99)	**0.006**

	Cardiovascular Hospitalization	
**Characteristics**	**HR (95% CI)**	**P-value**
Clinical		
Atrial Fibrillation	3.77 (1.54-9.23)	**0.004**
Anticoagulants	0.28 (0.13-0.58)	**0.001**
Diastolic BP per 10	0.66 (0.44-0.99)	**0.043**
Biochemical		
eGFR	0.96 (0.93-0.99)	**0.042**

Figure S1. Baseline and 1-year follow-up systolic and diastolic blood pressure for patients treated and untreated with tafamidis disease-modifying therapy. Observations: baseline (n=67 for tafamidis, n=41 for non-tafamidis) and 1-year follow-up (n=51 for tafamidis, n=18 for non-tafamidis).

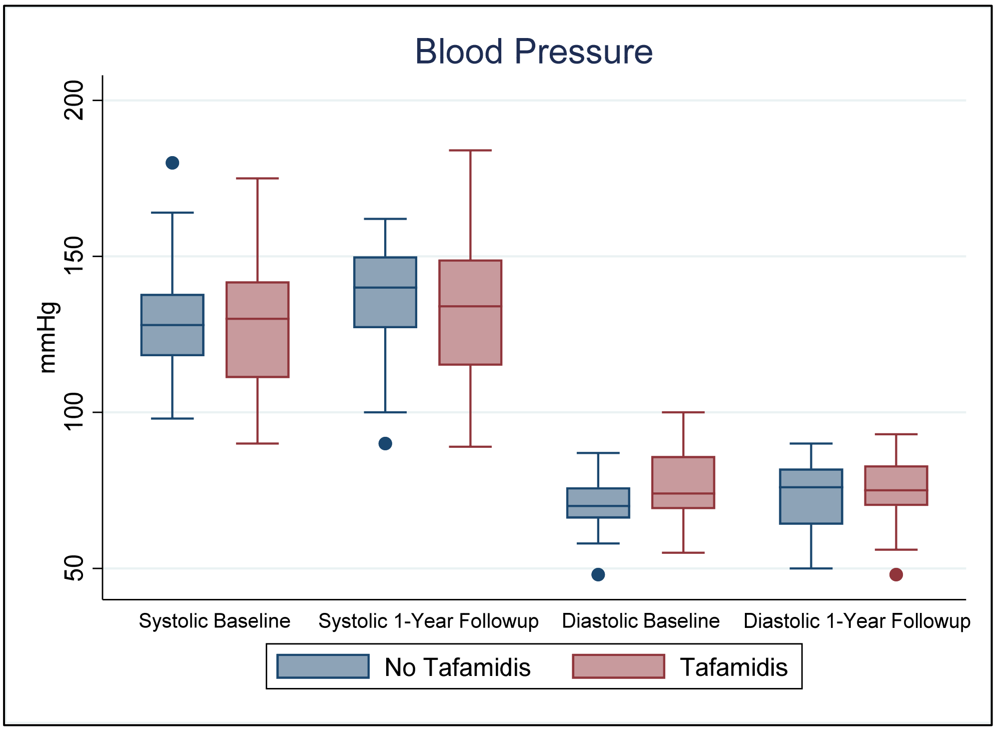

Supplement: Supplementary file 1 [file jcm-13-05490-s001.zip › jcm-3178404-supplementary.pdf]
